# 2-(5-Amino-2*H*-tetra­zol-2-yl)acetic acid

**DOI:** 10.1107/S1600536812014389

**Published:** 2012-04-06

**Authors:** Guo-Chun Zhang, Cheng-Fang Qiao, Chun-Sheng Zhou, Zheng-Qiang Xia

**Affiliations:** a Department of Chemistry and Chemical Engineering, Shaanxi Key Laboratory of Comprehensive Utilization of Tailing Resources, Shangluo University, Shangluo 726000, Shaanxi, People’s Republic of China; b College of Chemistry and Materials Science, Northwest University, Xi’an 710069, Shaanxi, People’s Republic of China

## Abstract

In the title mol­ecule, C_3_H_5_N_5_O_2_, the tetra­zole ring and carboxyl group form a dihedral angle of 82.25 (14)°. In the crystal, adjacent mol­ecules are linked through O—H⋯N, N—H⋯O and N—H⋯N hydrogen bonds into layers parallel to the *bc* plane.

## Related literature

For background to tetra­zole compounds, see: Zhao *et al.* (2008 ▶). For the use of 5-amino­tetra­zole-1-acetic acid in coord­ination chemistry, see: Li *et al.* (2010 ▶); Shen *et al.* (2011 ▶); Yang *et al.* (2008 ▶). For the crystal structures of similar compounds, see: Bryden (1956 ▶); Klapötke *et al.* (2009 ▶). For bond-length data, see: Allen *et al.* (1987 ▶).
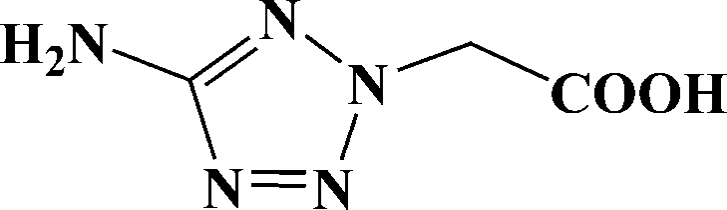


## Experimental

### 

#### Crystal data

C_3_H_5_N_5_O_2_
*M* *_r_* = 143.12
Monoclinic, 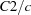

*a* = 18.381 (4) Å
*b* = 4.4429 (9) Å
*c* = 14.846 (3) Å
β = 90.850 (3)°
*V* = 1212.2 (4) Å^3^
*Z* = 8
Mo *K*α radiation
μ = 0.13 mm^−1^
*T* = 296 K
0.28 × 0.19 × 0.15 mm

#### Data collection

Bruker APEXII CCD diffractometer
Absorption correction: multi-scan (*SADABS*; Bruker, 2008 ▶) *T*_min_ = 0.970, *T*_max_ = 0.980
3040 measured reflections
1193 independent reflections
890 reflections with *I* > 2σ(*I*)
*R*_int_ = 0.024

#### Refinement

*R*[*F* ^2^ > 2σ(*F* ^2^)] = 0.044
*wR*(*F* ^2^) = 0.124
*S* = 1.03
1193 reflections
92 parameters
H-atom parameters constrained
Δρ_max_ = 0.21 e Å^−3^
Δρ_min_ = −0.19 e Å^−3^

### 

Data collection: *APEX2* (Bruker, 2008 ▶); cell refinement: *SAINT* (Bruker, 2008 ▶); data reduction: *SAINT*; program(s) used to solve structure: *SHELXS97* (Sheldrick, 2008 ▶); program(s) used to refine structure: *SHELXL97* (Sheldrick, 2008 ▶); molecular graphics: *ORTEP-3* (Farrugia, 1997 ▶); software used to prepare material for publication: *SHELXL97*.

## Supplementary Material

Crystal structure: contains datablock(s) I, global. DOI: 10.1107/S1600536812014389/cv5277sup1.cif

Structure factors: contains datablock(s) I. DOI: 10.1107/S1600536812014389/cv5277Isup2.hkl

Supplementary material file. DOI: 10.1107/S1600536812014389/cv5277Isup3.cml

Additional supplementary materials:  crystallographic information; 3D view; checkCIF report

## Figures and Tables

**Table 1:** Hydrogen-bond geometry (Å, °)

*D*—H⋯*A*	*D*—H	H⋯*A*	*D*⋯*A*	*D*—H⋯*A*
N5—H5*B*⋯O1^i^	0.86	2.36	3.080 (3)	141
N5—H5*A*⋯N4^ii^	0.86	2.23	3.064 (3)	163
O2—H2⋯N1^iii^	0.82	1.85	2.665 (2)	172

Symmetry codes: (i) 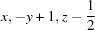; (ii) 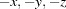; (iii) 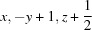.

**Fig. 1.**
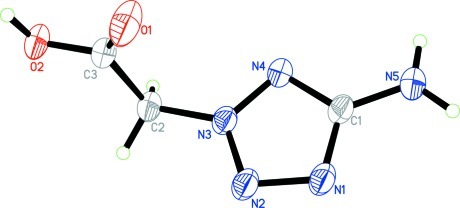
The molecular structure of (I) showing the atom numbering scheme and 30% probability displacement ellipsoids.

## supplementary crystallographic information

### Comment

In recent years, numerous tetrazole ligands were used to construct coordination compounds, which have been the subject of an intense research effort owing to their unique structures and potential applications in advanced materials (Zhao *et al.*, 2008). However, the study of coordination compounds with disubstituted tetrazole ligands is comparatively scarce. Recently, Li *et al.* (2010) reported a series of lanthanide-based compounds with 5-aminotetrazole-1-acetic acid, which possesses intriguing topological structures and predominant optics performance. Inspired by this interesting work, we report here the crystal structure of a 2,5-disubstituted tetrazole compound, (5-Amino-2*H*-tetrazole-2-yl)acetic acid, (I).

As shown in Fig. 1, the tetrazole ring (C1/N1—N4) is essentially planar with an r.m.s. deviation of 0.008 °. The carboxyl group (O1/C3/O2) and tetrazole ring are inclined at a dihedral angle of 82.25 (14) °. All bond lengths and angles are in the normal ranges (Allen *et al.*, 1987). The torsion angles N4–N3–C2–C3 = -80.6 (2) °, N2–N3–C2–C3 = 101.8 (2) °, O1–C3–C2–N3 = -3.6 (3) °, O2–C3–C2–N3 = 178.80 (17) °. In the crystal structure, intermolecular O—H···N, N—H···O and N—H···N hydrogen bonds (Table 1) link the molecules into a two-dimensional framework parallel to the *bc* plane.

### Experimental

The compound was obtained commercially (Aldrich). Colourless crystals suitable for the X-ray diffraction study were obtained by slow evaporation of an ethanol/water (2:1 *v*/*v*) solution of the compound (I) at room temperature.

### Refinement

All H atoms were placed in geometrically idealized positions, with C—H = 0.97 Å, N—H = 0.86 Å and O—H = 0.82 Å, and were thereafter treated as riding with *U*_iso_(H) = 1.2*U*_eq_(C,*N*) or 1.5*U*_eq_(O).

### Crystal data

**Table:**

C_3_H_5_N_5_O_2_	*F*(000) = 592
*M**_r_* = 143.12	*D*_x_ = 1.568 Mg m^−^^3^*D*_m_ = 1.568 Mg m^−^^3^*D*_m_ measured by not measured
Monoclinic, *C*2/*c*	Mo *K*α radiation, λ = 0.71073 Å
Hall symbol: -C 2yc	Cell parameters from 734 reflections
*a* = 18.381 (4) Å	θ = 3.5–22.2°
*b* = 4.4429 (9) Å	µ = 0.13 mm^−^^1^
*c* = 14.846 (3) Å	*T* = 296 K
β = 90.850 (3)°	Block, colourless
*V* = 1212.2 (4) Å^3^	0.28 × 0.19 × 0.15 mm
*Z* = 8	

### Data collection

**Table:**

Bruker APEXII CCD diffractometer	1193 independent reflections
Radiation source: fine-focus sealed tube	890 reflections with *I* > 2σ(*I*)
Graphite monochromator	*R*_int_ = 0.024
φ and ω scans	θ_max_ = 26.0°, θ_min_ = 2.2°
Absorption correction: multi-scan (*SADABS*; Bruker, 2008)	*h* = −22→22
*T*_min_ = 0.970, *T*_max_ = 0.980	*k* = −5→4
3040 measured reflections	*l* = −13→18

### Refinement

**Table:**

Refinement on *F*^2^	Primary atom site location: structure-invariant direct methods
Least-squares matrix: full	Secondary atom site location: difference Fourier map
*R*[*F*^2^ > 2σ(*F*^2^)] = 0.044	Hydrogen site location: inferred from neighbouring sites
*wR*(*F*^2^) = 0.124	H-atom parameters constrained
*S* = 1.03	*w* = 1/[σ^2^(*F*_o_^2^) + (0.0657*P*)^2^ + 0.3572*P*] where *P* = (*F*_o_^2^ + 2*F*_c_^2^)/3
1193 reflections	(Δ/σ)_max_ = 0.002
92 parameters	Δρ_max_ = 0.21 e Å^−^^3^
0 restraints	Δρ_min_ = −0.19 e Å^−^^3^

### Special details

**Table:**

Geometry. All e.s.d.'s (except the e.s.d. in the dihedral angle between two l.s. planes) are estimated using the full covariance matrix. The cell e.s.d.'s are taken into account individually in the estimation of e.s.d.'s in distances, angles and torsion angles; correlations between e.s.d.'s in cell parameters are only used when they are defined by crystal symmetry. An approximate (isotropic) treatment of cell e.s.d.'s is used for estimating e.s.d.'s involving l.s. planes.
Refinement. Refinement of *F*^2^ against ALL reflections. The weighted *R*-factor *wR* and goodness of fit *S* are based on *F*^2^, conventional *R*-factors *R* are based on *F*, with *F* set to zero for negative *F*^2^. The threshold expression of *F*^2^ > σ(*F*^2^) is used only for calculating *R*-factors(gt) *etc*. and is not relevant to the choice of reflections for refinement. *R*-factors based on *F*^2^ are statistically about twice as large as those based on *F*, and *R*- factors based on ALL data will be even larger.

### Fractional atomic coordinates and isotropic or equivalent isotropic displacement parameters (Å^2^)

**Table:**

	*x*	*y*	*z*	*U*_iso_*/*U*_eq_	
O2	0.17856 (8)	0.1746 (3)	0.30324 (8)	0.0527 (4)	
H2	0.1626	0.2644	0.3470	0.079*	
N2	0.18104 (10)	0.4658 (4)	0.01590 (11)	0.0493 (5)	
N3	0.15090 (8)	0.2586 (4)	0.06465 (10)	0.0414 (4)	
C3	0.14844 (11)	0.2824 (5)	0.22938 (12)	0.0426 (5)	
N4	0.08911 (9)	0.1468 (4)	0.03036 (11)	0.0485 (5)	
N1	0.13778 (10)	0.4994 (4)	−0.05556 (11)	0.0500 (5)	
C2	0.18346 (11)	0.1507 (5)	0.14771 (12)	0.0449 (5)	
H2A	0.2348	0.2010	0.1487	0.054*	
H2B	0.1793	−0.0668	0.1499	0.054*	
O1	0.10122 (10)	0.4680 (4)	0.22631 (10)	0.0752 (6)	
C1	0.08229 (11)	0.3035 (5)	−0.04569 (13)	0.0479 (5)	
N5	0.02757 (11)	0.2722 (6)	−0.10567 (13)	0.0787 (8)	
H5A	−0.0064	0.1434	−0.0962	0.094*	
H5B	0.0264	0.3809	−0.1536	0.094*	

### Atomic displacement parameters (Å^2^)

**Table:**

	*U*^11^	*U*^22^	*U*^33^	*U*^12^	*U*^13^	*U*^23^
O2	0.0661 (10)	0.0609 (10)	0.0308 (8)	0.0097 (8)	−0.0064 (6)	−0.0009 (6)
N2	0.0611 (10)	0.0547 (11)	0.0320 (9)	−0.0062 (9)	−0.0015 (7)	0.0015 (7)
N3	0.0464 (9)	0.0479 (10)	0.0300 (8)	0.0011 (7)	0.0009 (7)	−0.0019 (7)
C3	0.0479 (11)	0.0467 (12)	0.0329 (10)	0.0005 (10)	−0.0036 (8)	−0.0002 (8)
N4	0.0490 (9)	0.0622 (11)	0.0341 (9)	−0.0039 (8)	−0.0029 (7)	0.0042 (8)
N1	0.0604 (11)	0.0588 (12)	0.0309 (9)	−0.0029 (9)	−0.0027 (7)	0.0027 (7)
C2	0.0506 (11)	0.0502 (12)	0.0337 (10)	0.0068 (10)	−0.0047 (8)	0.0013 (8)
O1	0.0878 (12)	0.0979 (14)	0.0398 (9)	0.0459 (11)	−0.0038 (8)	−0.0048 (8)
C1	0.0483 (11)	0.0651 (14)	0.0303 (10)	0.0030 (10)	0.0015 (8)	0.0015 (9)
N5	0.0607 (12)	0.125 (2)	0.0503 (12)	−0.0216 (12)	−0.0165 (9)	0.0320 (12)

### Geometric parameters (Å, º)

**Table:**

O2—C3	1.312 (2)	N4—C1	1.331 (3)
O2—H2	0.8200	N1—C1	1.351 (3)
N2—N3	1.300 (2)	C2—H2A	0.9700
N2—N1	1.325 (2)	C2—H2B	0.9700
N3—N4	1.334 (2)	C1—N5	1.341 (2)
N3—C2	1.444 (2)	N5—H5A	0.8600
C3—O1	1.198 (2)	N5—H5B	0.8600
C3—C2	1.500 (3)		
			
C3—O2—H2	109.5	N3—C2—H2A	109.1
N3—N2—N1	105.66 (16)	C3—C2—H2A	109.1
N2—N3—N4	114.75 (16)	N3—C2—H2B	109.1
N2—N3—C2	122.39 (16)	C3—C2—H2B	109.1
N4—N3—C2	122.81 (17)	H2A—C2—H2B	107.8
O1—C3—O2	125.45 (18)	N4—C1—N5	124.7 (2)
O1—C3—C2	123.87 (18)	N4—C1—N1	111.56 (17)
O2—C3—C2	110.64 (17)	N5—C1—N1	123.72 (19)
C1—N4—N3	101.42 (17)	C1—N5—H5A	120.0
N2—N1—C1	106.60 (16)	C1—N5—H5B	120.0
N3—C2—C3	112.53 (16)	H5A—N5—H5B	120.0
			
N1—N2—N3—N4	0.2 (2)	O1—C3—C2—N3	−3.6 (3)
N1—N2—N3—C2	177.93 (17)	O2—C3—C2—N3	178.80 (17)
N2—N3—N4—C1	−0.1 (2)	N3—N4—C1—N5	179.7 (2)
C2—N3—N4—C1	−177.82 (17)	N3—N4—C1—N1	0.0 (2)
N3—N2—N1—C1	−0.2 (2)	N2—N1—C1—N4	0.2 (2)
N2—N3—C2—C3	101.8 (2)	N2—N1—C1—N5	−179.6 (2)
N4—N3—C2—C3	−80.6 (2)		

### Hydrogen-bond geometry (Å, º)

**Table:**

*D*—H···*A*	*D*—H	H···*A*	*D*···*A*	*D*—H···*A*
N5—H5*B*···O1^i^	0.86	2.36	3.080 (3)	141
N5—H5*A*···N4^ii^	0.86	2.23	3.064 (3)	163
O2—H2···N1^iii^	0.82	1.85	2.665 (2)	172

Symmetry codes: (i) *x*, −*y*+1, *z*−1/2; (ii) −*x*, −*y*, −*z*; (iii) *x*, −*y*+1, *z*+1/2.
